# Metabolic disturbances are associated with psychiatric readmission: results from a Swiss psychiatric cohort

**DOI:** 10.1192/j.eurpsy.2022.217

**Published:** 2022-09-01

**Authors:** N. Laaboub, M. Gholam, C. Dubath, C. Grosu, M. Piras, K. Von Plessen, A. Von Gunten, P. Conus, M. Preisig, C. Eap

**Affiliations:** Lausanne University Hospital, Psychiatry, Prilly, Switzerland

**Keywords:** readmission, psychiatry, metabolic disturbances

## Abstract

**Introduction:**

High BMI has been associated with psychiatric rehospitalisation.

**Objectives:**

We aimed to replicate this finding in a large Swiss psychiatric cohort and to examine whether other metabolic disturbances are independently associated with psychiatric readmission.

**Methods:**

Data on 16’727 hospitalizations of 7’786 patients admitted between January 1^st^, 2007 and December 31^st^, 2019 at the Department of Psychiatry of the Lausanne University Hospital, were collected. Metabolic syndrome was defined according to International Diabetes Federation definition. Generalized Linear Mixed Models were used to investigate the associations between psychiatric readmission and metabolic syndrome and/or its five components.

**Results:**

The readmitted population (N=2’935; 37.7% patients) had higher BMI, and were more likely to have central obesity, hypertriglyceridemia, and hypertension. Multivariate analyses confirmed that having a BMI ≥ 25 kg.m^-2^ was associated with psychiatric readmission (25 kg.m^-2^≤ BMI< 30 kg.m^-2^: OR = 1.88; 95%CI [1.55-2.29]; BMI≥30 kg.m^-2^: OR = 3.5; 95%CI [2.85-4.30]) when compared to patients with 18.5≤BMI<25 kg.m^-2^. Interestingly, novel factors associated with readmission were identified including metabolic syndrome (OR = 1.57, 95%CI [1.05-2.33]), central obesity (OR = 1.81, 95%CI [1.33-2.46]), hypertriglyceridemia (OR = 1.59; 95%CI [1.38-1.83]), HDL hypocholesterolemia (OR = 1.22; 95%CI [1.06-1.40]) and hyperglycemia (OR = 1.58; 95%CI [1.35-1.85]).

**Conclusions:**

Metabolic syndrome, central obesity, hypertriglyceridemia, HDL hypocholesterolemia, hyperglycemia and obesity were associated with psychiatric readmission. Possible causes will be presented and discussed (e.g. reduced adherence to treatment in patients with metabolic disorders, multiple psychotropic treatments in non-responders increasing the risk of metabolic worsening).

**Disclosure:**

No significant relationships.

